# Interventional strategies for pulmonary arterial hypertension: Current perspectives and future directions

**DOI:** 10.1038/s41440-026-02660-2

**Published:** 2026-05-19

**Authors:** Konstantinos Platanias, Stergios Soulaidopoulos, Marios Sagris, Athanasios Makris, Sofia Touriki, Alexis Giannakodimos, Costas Thomopoulos, Konstantinos Tsioufis

**Affiliations:** 1 National and Kapodistrian University of Athens, Medical school, Athens, Greece; 2 Hippokration General Hospital, National and Kapodistrian University of Athens, School of Medicine, Athens, Greece; 3 First Cardiology department, National and Kapodistrian University of Athens, Hippokrateion hospital, Athens, Greece; 4 Laiko General Hospital of Athens, Athens, Greece; 5 1st Department of Cardiology, Medical School of Athens University, Hippokration Hospital, Athens, Greece

**Keywords:** Pulmonary arterial hypertension, Pulmonary artery denervation, Balloon atrial septostomy, Renal denervation, Transcatheter Potts shunt

## Abstract

Pulmonary arterial hypertension (PAH) often leads to right heart failure and death. While pharmacological therapies offer symptomatic relief and functional improvement, many patients remain refractory or progress despite optimal treatment. This review explores emerging interventional strategies besides pharmacotherapy. Techniques such as pulmonary artery denervation (PADN), balloon atrial septostomy (BAS), renal denervation, transcatheter Potts shunt, right ventricular assist devices, catheter-based cell therapies and implantable hemodynamic monitoring devices are evaluated based on safety, efficacy, and long-term outcomes. Notably, PADN has demonstrated encouraging results across multiple trials, with improvements in exercise capacity, hemodynamic parameters, and clinical status. BAS remains a palliative bridge to transplantation in severe cases, with newer modifications aiming to overcome spontaneous closure. Renal denervation and transcatheter Potts shunts show promise but require further validation. Catheter-based cell therapies show promising results and could perhaps be used as an alternative in the future. Moreover, mechanical support devices like ECMO and pressure monitors such as CardioMEMS offer adjunctive benefits in advanced disease. Right ventricular assist devices could in circumstances be used as bridge to transplant or bridge to recovery. This review illustrates the need for robust randomized controlled trials in PAH.

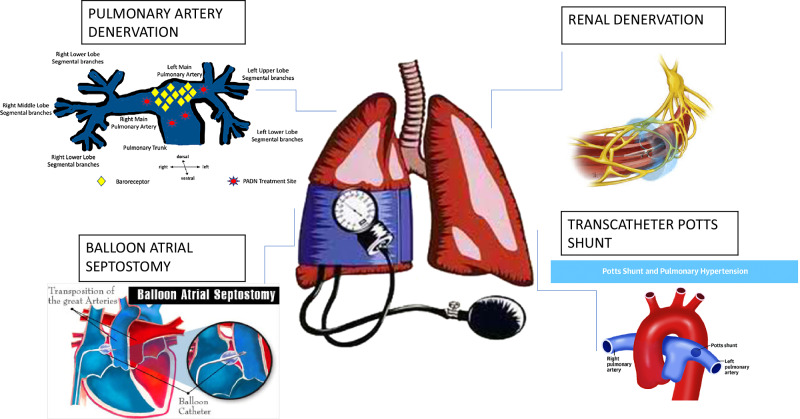

## Highlights

- Pulmonary arterial hypertension is a progressive and life-threatening disease that commonly culminates in right heart failure and premature mortality.
- Despite advances in targeted pharmacotherapy, a significant proportion of patients remain treatment-refractory or continue to deteriorate.
- Emerging interventional strategies—including pulmonary and renal denervation, balloon atrial septostomy, and the Potts shunt—represent promising adjunctive options for advanced disease.

## Introduction

Pulmonary Hypertension (PH) affects 1% of the population globally. Pulmonary arterial hypertension (PAH) refers to the first group of PH, according to the ESC classification, and affects all ages, mainly those between 30 and 60 years old. It includes idiopathic and heritable forms, as well as cases associated with drugs and toxins, connective tissue disorders, HIV infection, portal hypertension, congenital heart disease, and schistosomiasis. Additionally, it encompasses conditions with venous or capillary involvement and persistent pulmonary hypertension of the newborn. The most common cause of PH is left heart disease (Group 2), followed by lung disease (Group 3). Pulmonary hypertension is defined as a mean pulmonary arterial pressure >20 mmHg. Regarding Group 1 is localized in the precapillary vessels and is characterized by mean pulmonary arterial pressure >20 mmHg, pulmonary capillary wedge pressure or left ventricular end diastolic pressure <15 mmHg and pulmonary vascular resistance >3 Wood Units. Pulmonary vasodilators that regulate the prostacyclin, endothelin and nitric oxide pathways represent the approved medical therapies for PAH for the moment. However, a substantial number of patients will not sufficiently respond to pharmaceutical treatment or will need additional therapeutic measures after an initial period of response. Risk stratification and early combination pharmacotherapy have been recently proposed by the ESC/ERS Guidelines, but in few cases there are still patients who do not respond to these measures. Lung transplantation is another option for non-responders and patients who do not tolerate medical treatment, but donor availability is limited, and even after transplantation, survival ranges from seven to ten years [1–5].

To address this need, further treatment options for pulmonary hypertension must be explored. Interventional cardiology presents a promising avenue, offering patients the possibility of prolonged survival until transplantation or improved clinical and functional status when transplantation is not feasible. In this context, several interventional techniques have been proposed and tested for the treatment of PAH; however, current evidence remains limited. The aim of this article is to summarize the available data on interventional techniques and options for PAH management, making clear that their clinical impact is not yet established, and further research is needed before some of these techniques be part of clinical practice [6–8]

### Pulmonary artery denervation (PADN) for pulmonary arterial hypertension

Denervation is a technique that aims to destroy the sympathetic nerves of the artery using variable types of energy. Apart from disrupting the sympathetic nervous system, PADN leads to pulmonary vasodilation, improved hemodynamics and many studies suggest that PADN leads to upregulation of beta-adrenoceptors and downregulation of detrimental alpha-adrenoceptors. PADN inhibits the nerves that promote smooth muscle proliferation, leading to decreased vascular remodeling. Moreover, regulation of renin-angiotensin-aldosterone system decreases pulmonary vascular resistance and reduces vascular remodeling. The most experience about the technique comes from renal denervation, where the sympathetic denervation of the renal arteries have been found to substantially reduce the blood pressure in patients with resistant hypertension. However, the exact mechanisms of PADN remain unknown and the technique is only used for investigational purposes [9–12].

The first-in-man study, Pulmonary Artery Denervation 1 (PADN-1) study, was conducted by Chen et al. in 2013 with a one year duration. The purpose of the study was to evaluate the safety and efficacy of pulmonary artery denervation (PADN) in patients with idiopathic pulmonary arterial hypertension, who were not optimally responding to medical treatment. Mean PAP was adequately reduced (from 55 ± 5 mmHg to 36 ± 5 mmHg, *p* < 0.01), 6MWT was improved (from 324 ± 21 m to 491 ± 38 m, *p* < 0.006), as well as the Tei index (from 0.3 ± 0.04 to 0.50 ± 0.04, *p* < 0.001), compared to the control group. The clinical follow-up reported no PA perforation/dissection, aneurysm or acute thrombus formation. None of the patients needed lung transplantation during follow-up neither re-hospitalization compared to control group (62.5%, 5 re-hospitalizations). Low mortality rates were observed with only 2 deaths, one in each group [13].

The PADN-5 study demonstrated that PADN increases 6MWT for patients with combined Pre- and Post-Capillary Pulmonary Hypertension associated with left heart failure. 6 months later, there was an increase of 83 m in the PADN group versus 15 m in the sildenafil group and PADN group had a significant lower Pulmonary Vascular Resistance (PVR) than the sildenafil group, while symptoms worsening was more frequent in the second than in the first group. Consequently, PADN treatment showed hemodynamic and clinical improvement in patients with with Pre- and Post-Capillary Pulmonary Hypertension [14]. The same team of scientists, collected data 3 years after the PADN-5 trial. The rate of rehospitalization, the worsening of symptoms, the 6-MWT and NTpro-BNP were all better in the PADN group than in the sildenafil group [15].

The TROPHY-1(Treatment of Pulmonary Hypertension 1) trial was published in 2020, concerning the intravascular ultrasound PADN to treat PAH. This was a multicenter, international, open-label trial of 8 different specialist centers. The results showed that there were no procedure-related serious adverse events. In contrast to previous studies, there was not an acute change in pulmonary hemodynamics. At 4 or 6 months, PVR was reduced at about 17.8%, while the 6MWT increased by 42 m. Quality-of-life score, NT-proBNP, heart rate and systemic blood pressure did not change [16].

In 2022, Zhang et al. published the PADN-CFDA trial trying to evaluate the use of PADN in PAH. The group A received PADN plus a phosphodiesterase-5 inhibitor, and the second group received a sham procedure plus a phosphodiesterase-5 inhibitor. Results showed positive effects of PADN in 6MWD, PVR, right ventricular function, NT-proBNB and on clinical worsening 6 months later [17]. Similar findings were observed when the same research team reassessed the initial patient cohort one year later. Specifically, clinical worsening occurred in 4.8% of patients in the first group compared to 23.1% in the second group. PAH-related hospitalizations were also more frequent in the second group. Additionally, the 6MWD remained higher in the first group by 82 m at the one-year follow-up [18].

### Balloon atrial septostomy (BAS) for pulmonary arterial hypertension

BAS needs femoral venous vascular access followed by trans-catheter delivery of a septal crossing apparatus. Using echocardiography, either transesophageal or intracardiac, transseptal puncture is performed, and then a wire is advanced into the left superior pulmonary vein and a catheter is advanced into the left atrium, while graded balloon inflation is then performed [19, 20]. BAS could improve hemodynamic status and clinical symptoms by establishing an atrial-level shunt. It is the oldest interventional approach of pulmonary hypertension while the first attempted was performed by Rich and Lam in 1983. Despite that fact, it is clear that BAS is rarely used in PAH and is currently restricted to high risk patients, including those who have been waiting for lung transplantation or those whose response to maximal medical therapy is insufficient, unavailable or not well tolerated [11]. Between 1991 and 2016, a total of 16 studies encompassing 204 patients yielded noteworthy findings. Among them, nine studies reported data on right atrial pressure, all demonstrating improved outcomes with BAS. Similarly, 14 studies assessed cardiac index, with all indicating an increase following BAS. Across all 16 studies, arterial oxygen saturation was consistently lower in the BAS group. Additionally, nine studies documented cases of spontaneous septostomy closure. Overall, the findings suggest that while BAS offers high postprocedural and short-term survival rates, its less favorable long-term survival outcomes reinforce its role as a bridge to transplantation [21].

In 2022, Yan et al. published the first-in-human modified atrial septostomy, which included radiofrequency ablation and balloon dilation. Among 19 patients who participated in the study between 2018 and 2021, ten of them had idiopathic PAH and nine had PAH related to repaired congenital heart disease. After the intervention, the mean right atrial pressure was decreased, the systolic aortic pressure and cardiac index were increased, while the Sa0_2_ was mildly decreased. Only one patient had increased pericardiac effusion, which was treated with pericardiocentesis. The mid-term follow up (15,5 months later) showed that, all interatrial communications were patent and well functioned and that the NYHA class was increased by 1, revealing the improvement of patients symptoms [20].

### Transcatheter potts shunt (TPS)

Potts shunt concerns the communication between the left pulmonary artery and the descending aorta. Potts shunt is mainly used in children with severe pulmonary hypertension refractory to medical therapy. This procedure, connecting the pulmonary artery with the descending aorta, protects from a paradoxic emboli, as it separates the brain and the myocardium from the desaturated blood. Surgical Potts shunt includes all the dangers of a surgery. TPS is still used rarely and in high risk patients. High risk includes patients with end-stage pulmonary arterial hypertension, who are ineligible for traditional surgery and have a high probability of perioperative mortality. In other words, patients who remain symptomatic even at rest, patients with frequent syncope episodes, who do not respond on maximum drug combination therapy and those who are currently stabilized on extracorporeal membrane oxygenation as a bridge to intervention, are all in a high risk status [11, 22–24].

In 2013, Ventura et al. attempted a catheter-based Potts shunt in seven patients with severe pulmonary arterial hypertension and right ventricular failure refractory to medical therapy. However, three patients decompensated and died before the intervention, highlighting the high mortality in this population. The remaining four underwent transcatheter shunt creation with stent placement between the great vessels. Two patients survived for at least four months with symptomatic improvement, while one died five days post-procedure due to multisystem organ failure, complicated by preexisting pneumonia. The fourth patient suffered a fatal intrathoracic bleed during the procedure [25].

In 2016, Boudjemline et al. assessed the safety and efficacy of transcatheter Potts shunt in children. TPS was performed with radiofrequency energy for vessel perforation and deployment of a covered stent, in order to connect the left pulmonary artery and the descending aorta. Results showed that TPS was successfully performed in 6 children with severe PAH and dysfunction of right ventricle. Two of them, with pre-existing severe biventricular dysfunction and pericardial effusion, were led to a low preload of left ventricle, which had the result of cardiac arrest, brain damage and death. In one patient, the stent was dislodged and embolized the iliac artery, but that was corrected with a second stent. The other 4 patients were alive after a follow-up of about 10 months. Noticeably, in 3 patients, intravenous vasodilator therapy stopped after the TPS [26].

In 2022, Haddad et al. evaluated the long-term outcomes of transcatheter Potts shunt (TPS) in patients with severe pulmonary arterial hypertension (PAH). TPS was performed either through endovascular intervention on a patent arterial duct or via radiofrequency perforation with stent placement (ductal TPS). Among the 13 patients, an improvement in functional class was observed at follow-up; however, no significant reduction in medication use or NT-proBNP levels was noted. The median follow-up duration was 77.4 months, with a survival rate of 100% at one year and 92.3% at six years. Notably, re-do procedure required in most of the patients [27]. Interestingly, Mirabile et al. investigated the use of extracorporeal membrane oxygenation (ECMO) in selected patients immediately following TPS intervention. Their findings suggested that while ECMO may provide temporary support in cases of cardiac arrest, its benefit is primarily limited to managing hemodynamic instability resulting from the TPS procedure [28].

### Renal denervation for pulmonary arterial hypertension

It is known that sympathetic nervous system and renin-angiotensin-aldosterone system are associated with pulmonary hypertension. Thus, renal denervation could potentially decrease the activity of renin-angiotensin-aldosterone system and limit down the pulmonary hypertension. Renal denervation in PAH remains experimental and has thus far been evaluated primarily in preclinical and animal studies. Investigating its potential role in PAH may help clarify the underlying mechanisms involved and inform future therapeutic perspectives [2, 11, 29].

In 2015 Qingyan et al. examined the impact of renal sympathetic denervation in pulmonary hypertension. They used 22 beagles and made three groups. The first one was the control group, the second developed pulmonary hypertension and the third one was the group that developed pulmonary hypertension and was submitted to renal denervation. 8 weeks after the injection of substances that provoked pulmonary hypertension, results showed that hemodynamic parameters were better in the renal denervation group than in the pulmonary hypertension group. The levels of aldosterone and B-type natriuretic peptide were higher in the 2 groups of pulmonary hypertension-induced groups than in the control group, but pulmonary hypertension group had higher levels of them than the renal denervation group [30].

In 2017 Liu et al. using rats showed that renal denervation improved pulmonary vascular remodeling, decreased right ventricular afterload and right ventricular stiffness. Consequently, the hypothesis that neurohormonal dysfunction, including activation of sympathetic nervous system and renin-angiotensin-aldosterone system, play an important role in pulmonary hypertension, is confirmed [31]. However, in 2021, Vakhrushev et al. investigated the impact of renal denervation (RDN) on systemic and pulmonary hemodynamics in a swine model. Notably, no significant differences were observed between the RDN and sham groups [32].

### Catheter-based cell therapies

As far as catheter-based therapies are concerned, endothelial progenitor cells (EPCs) or cardiac-derived cells (CDCs) could directly be delivered into the pulmonary arteries, leading to better hemodynamic and clinical status of the patient. In human studies with EPCs, there was observed reduction in pulmonary arterial pressure and pulmonary vascular resistance, increase of cardiac output, while Wang et al. showed improvement in 6-min walk test and Zhu et al. found better NYHA class of the patients [33]. Lewis et al. used cardiac-derived cells, also known as cardiosphere-derived cells, showing a safe profile without adverse events, improvement in 6-min walk test and right ventricle function and neutral impact on pulmonary arterial pressure and pulmonary vascular resistance. Current studies in rats have been conducted, but further studies in clinical level are needed so that catheter-based therapies could be used in the future as an alternative in PAH patients [34].

### Implantable pulmonary artery pressure monitoring devices and mechanical circulatory support

Right heart catheterization does not allow for frequent monitoring of right ventricular function. Theoretically, those implantable devices could earlier explore hemodynamic changes and contribute to therapy optimization. Apart from continuous monitoring, these devices could act not only for diagnostic purposes, but also for therapeutic. They could provide immediate hemodynamic stabilization in cases of cardiogenic shock and act as a bridge to recovery, bridge to transplant or bridge to decision method. However, the clinical benefit or superiority of these devices is not yet established and they could be used as alternatives [35]. The CardioMEMS HF System, an ambulatory implantable hemodynamic monitor, was initially studied in heart failure (HF) patients in the CHAMPION trial [36, 37]. Building on this, in 2019, Benza et al. evaluated its use in patients with PAH and NYHA class III or IV right-sided HF under treatment. Their findings demonstrated that the system was safe, with no device-related serious adverse events. One month post-implantation, PAP was reduced, and cardiac output improved, while after one year, patients showed better functional status [35]. Another study, in 2024, by Labrandero et al. showed that the CardioMEMS device is safe in pediatric population and allows the hemodynamic monitoring of patients with heart failure and pulmonary hypertension [38].

Considering the mechanical support, ECMO serves as a circulatory support system for right ventricular failure, playing a crucial role as a bridge to transplantation in patients with severe right heart failure. Additionally, it is utilized as a bridge to recovery in cases where right heart failure is potentially reversible [3]. In 2017, Salman et al. showed that in patients with severe PH, who are submitted to lung transplantation, the use of ECMO peri and post-operatively improved survival rates in 1 year follow up [39]. In 2018, Moser et al. evaluated the postoperative use of ECMO in patients undergoing bilateral lung transplantation for idiopathic PAH. Among 41 patients, those who received prolonged ECMO postoperatively demonstrated better survival rates than those who did not, highlighting its value as a supportive tool in this high-risk population [40]. Similarly, Stacel et al. (2020) reported that ECMO served as an effective postoperative left ventricular conditioning strategy in lung transplant recipients with PAH, improving cardiac function while minimizing adverse effects [41]. In their 5-year analysis, further demonstrated that lung transplant recipients, regardless of PAH status, had comparable survival rates and hospital stays when ECMO was used peri-operatively and postoperatively, despite PAH patients generally facing higher post-transplant complication risks [42]. Furthermore, ECMO could be used as a bridge to recovery in specific circumstances. In 2024, Pequignot et al. studied a case of a 30-year-old woman with heritable PAH. The patient remained hypoxemic despite the use of mechanical ventilation and fluid optimization. After the implantation of ECMO in the patient, epoprostenol infusion, bosentan and tadalafil were administered. A dramatic improvement was observed, decannulation was allowed 7 days after the pharmacological treatment, and 29 days later the patient was discharged from the hospital. More research has to be made, as far as this bridge to recovery strategy is concerned, identifying the patients who would mostly benefit from it [43] (Table 1).

**Table 1:** Summary of different interventional studies in pulmonary arterial hypertension

Technique	Study Name / Author	Year	Sample Size	Key Findings	Comments
**Pulmonary Artery Denervation (PADN)**	PADN-1 (Chen et al.)	2013	21 (13 PADN, 8 control)	↓ PAP (55 → 36 mmHg), ↑ 6MWT (324 → 491 m), improved Tei index	First-in-man study; safe and effective short-term
PADN	PADN-5 (Zhang et al.)	2019	98	↑ 6MWT by 83 m vs. 15 m (sildenafil), ↓ PVR	Clinical and hemodynamic improvement
PADN	PADN-5 Follow-up	2023	—	Better 6MWT, NT-proBNP, and rehospitalization rate after 3 years	Demonstrates long-term benefit
PADN	TROPHY-1 (Rothman et al.)	2020	Multicenter	PVR ↓ ~18%, ↑ 6MWT by 42 m	IVUS-guided PADN; no serious adverse events
PADN	PADN-CFDA (Zhang et al.)	2022	128	↑ 6MWD, ↓ PVR, improved NT-proBNP	RCT showing benefit over sham
PADN	PADN-CFDA 1-year (Kan et al.)	2023	—	↓ Clinical worsening (4.8% vs. 23.1%), ↑ 6MWD by 82 m	Benefits across all risk groups
**Balloon Atrial Septostomy (BAS)**	Khan et al. (Meta-analysis)	2019	204 (16 studies)	↑ Cardiac index, ↓ RA pressure, ↓ SaO₂; 9 studies with spontaneous closure	Effective short-term; bridge to transplantation
BAS	Yan et al. (CURB)	2022	19	Maintained shunt patency, ↓ RA pressure, ↑ CI, NYHA improved by 1 class	Modified technique with promising mid-term results
**Renal Denervation (RDN)**	Qingyan et al. (Beagles)	2015	22 (animal study)	↓ RV remodeling, ↓ aldosterone, ↓ BNP	Preclinical model; early intervention
RDN	Liu et al. (Rats)	2017	—	↓ Pulmonary vascular remodeling, ↓ RV stiffness	Supports neurohormonal hypothesis
RDN	Vakhrushev et al. (Swine)	2021	—	No hemodynamic benefit; increased arterial injury	Raises safety concerns
**Transcatheter Potts Shunt (TPS)**	Ventura et al.	2013	7	2 improved; 2 died pre-procedure; 1 procedural death	High-risk population; mixed outcomes
TPS	Boudjemline et al.	2017	6	4 survivors; ↓ IV therapy in 3	Procedural complications reported
TPS	Haddad et al.	2022	13	↑ NYHA class; no ↓ medications; 92.3% survival at 6 years	Long-term benefit; redo procedures common
TPS	Mirabile et al.	2021	—	ECMO useful in managing TPS complications	ECMO may stabilize post-TPS instability
**Implantable Monitors**	Benza et al. (CardioMEMS)	2019	—	Safe; ↓ PAP, ↑ CO, ↑ functional status at 1 year	Remote monitoring in PAH with RV failure
Implantable Monitors	Labrandero et al. (Pediatrics)	2024	—	Safe and feasible in children with PAH	Pediatric monitoring tool
**Mechanical Support (ECMO)**	Salman et al.	2017	—	ECMO improved 1-year survival post-transplant	Peri- and postoperative benefit
ECMO	Moser et al.	2018	41	Prolonged ECMO improved survival	Support in PAH lung transplantation
ECMO	Stacel et al.	2020–2024	—	ECMO aided LV conditioning; stable long-term outcomes	Confirms long-term feasibility
**Right Ventricular Assist Devices (RVADs)**	Punnoose et al.	2012	NA	↑ Cardiac output, ↓ RA pressure, ↑ pulmonary and capillary pressures; TR had no effect	Systemic benefit but risk of worsening pulmonary pressures
RVADs	Shavik et al.	2019	NA	Similar hemodynamic findings to Punnoose et al.	Confirms benefits and limitations
**Cell-Based Therapies**	Evans et al. (EPCs)	2021	NR	↓ PAP and PVR, ↑ CO, improved 6MWT and NYHA class	Functional and hemodynamic improvement
Cell-Based Therapies	Lewis et al. (CDCs)	2024	NR	Safe; ↑ 6MWT and RV function; neutral PAP and PVR	Promising but needs further clinical trials

(Summary of the interventional studies presenting for each technique the sample size of the study and the key findings of each of them) [1–45]

**Fig. 1:**
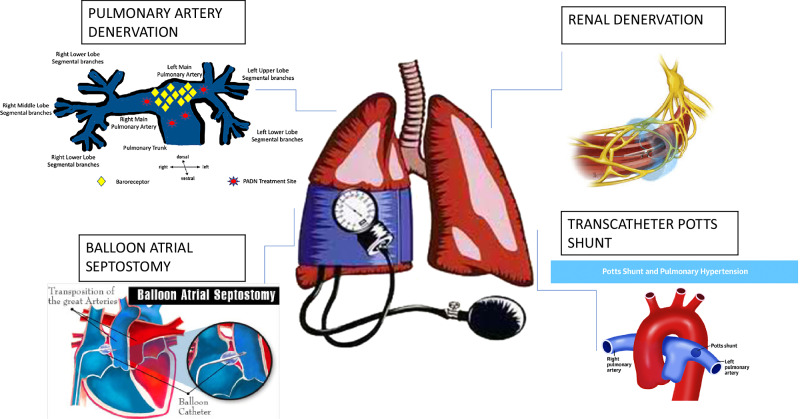
Interventional approaches in pulmomary arterial hypertension. Pulmonary artery denervation- destroying the sympathetic nerves of the artery, renal denervation- decreasing the activity of renin-angiotensin-aldosterone, balloon atrial septostomy-creating an atrial shunt and transcatheter Potts shunt-making a communication between the left pulmonary artery and the descending aorta, emerge as promising techniques in pulmonary arterial hypertension. Each technique has its own pros and cons and further investigation with randomized controlled trials is needed [1, 11]

In pulmonary arterial hypertension, right ventricular assist devices (RVADs) could mechanically support the right part of the heart. They could be used either as a bridge to transplant or as a bridge to recovery. Their use is accompanied by worsening pulmonary pressures, so careful selection of the patients is needed. Punnoose et al. conducted a theoretical analysis and found out that RVADs improved the cardiac output, decreased the right atrial pressure but raised pulmonary and capillary pressures. The presence of tricuspid regurgitation did not significantly affect the use of RVADs [44]. Similar hemodynamic findings were suggested by Shavik et al. [45] (Fig. 1).

## Conclusion

In conclusion, this study emphasizes the evolving role of interventional approaches in the management of pulmonary arterial hypertension (PAH). While each intervention presents distinct benefits and risks, careful patient selection by a multidisciplinary team at specialized centers is crucial to optimizing outcomes and minimizing adverse events. To better define the role of each intervention, future randomized controlled trials are essential for advancing our understanding and improving clinical management strategies for PAH.
